# Seroprevalence of human cytomegalovirus in blood donors

**DOI:** 10.3205/dgkh000621

**Published:** 2026-02-06

**Authors:** Abhishri Lakshmi Kamal Kanna, Aswin Manikandan Mathialagan, Subhashini Paari, Harish Mardawada

**Affiliations:** 1 Pathology Department, Sree Balaji Medical College and Hospital, Chennai, India; 2 Radiology Department, Sree Balaji Medical College and Hospital, Chennai, India

**Keywords:** cytomegaly, blood donors, seroprevalence, socioeconomic influence

## Abstract

**Introduction:**

Transfusions of blood are a vital component of medical treatment. On the other hand, infections linked to transfusions are possible risks. Blood collection, processing, and testing to ensure safety precautions are taken to reduce the chance of transfusion-transmitted illnesses are important.

The cytomegaly virus (CMV) is transfusion-transmitted (TT) and is a major global health concern. Seropositivity rates for CMV range from 60% to 100% in the population. Among the most important infections that infect people with immunosuppression is CMV.

The present study was conducted in response to the growing demand for CMV-free blood products and to determine the prevalence of CMV antibodies among consenting blood donors. In India, there is a paucity of research on this topic, and the present study is intended to help fill the resulting knowledge gap.

**Method:**

This prospective study was carried out in the blood bank of the Pathology Department of Sree Balaji Medical College in Chromepet, Chennai, from 2022 to 2024, after ethics approval was obtained. A total of 116 willing blood donors were chosen.

The tests were performed with a chemiluminescence kit for the detection of IgG and IgM.

**Results:**

Eighty-eight (88; 76.0%) of the 116 donors were men, and 28 (24%) were women. The blood donors’ ages were distributed as follows: 1.2% >40 years of age, 4.3% were 36 to 39, 8.2% were 31 to 35, 19.1% were 26 to 30, and 34.1% were 18 to 20 years of age. Seventy-five (75) people tested positive and 41 tested negative for IgG, yielding a 64.6% total CMV prevalence rate. In accordance with the CLIA regulations, none of the 116 blood donors had CMV IgM antibody reactivity. Individuals ≥26 years showed an increased prevalence of IgG positivity. Among the IgG serology-positive individuals, the number of males who showed positivity was 68 (64.76%), whereas only 7 females (63.63%) did so. 40% of IgG serology-positive donors had a high socioeconomic status [SES], 66.3% were of middle SES, and those donors of low SES were 100% IgG serology-positive.

**Conclusion:**

The prevalence points to an endemic infection, which may be related to factors such as age, gender, and socioeconomic status. IgM anti-CMV antibodies were absent in all donors. Therefore, because of the high cost and scarcity of IgM antibody-positive donors, immunoglobulin M anti-CMV antibody screening can be limited to high-risk recipients. Leucocyte-depleted cellular blood products and the selection of CMV-IgG and CMV-IgM-negative donors are two prophylactic approaches to lower the seroprevalence of transfusion-transmitted (TT)-CMV. Thus, our study highlights the importance of screening for CMV at blood banks, the need to assess risk factors for TT-CMV, and the need to take preventive action.

## Zusammenfassung

**Einleitung:**

Bluttransfusionen sind ein wichtiger Bestandteil der medizinischen Behandlung. Andererseits stellen Infektionen im Zusammenhang mit Transfusionen mögliche Risiken dar. Deshalb sind Sicherheitsvorkehrungen bei der Blutentnahme, -verarbeitung und -testung wichtig, um das Risiko von durch Transfusionen übertragbaren Krankheiten zu verringern.

Das Zytomegalie-Virus (CMV) wird durch Bluttransfusionen übertragen und stellt weltweit ein Gesundheitsproblem dar. Die Seropositivitätsraten für CMV liegen in der Bevölkerung zwischen 60% und 100%. Zu den wichtigsten Infektionen bei Immunsuppression gehören CMV.

Die Studie wurde als Reaktion auf die wachsende Nachfrage nach Cytomegalovirus-freien Blutprodukten und zur Bestimmung der Prävalenz von CMV-Antikörpern unter freiwilligenden Blutspendern durchgeführt, weil es in Indien nicht viele derartige Studien gibt.

**Methode:**

Die prospektive Studie wurde in der Blutbank der Pathologieabteilung des Sree Balaji Medical College in Chromepet, Chennai, von 2022 bis 2024 auf der Grundlage des positive Ethikvotums durchgeführt. Es wurden 116 freiwillige Blutspender eingeschlossen.

Die Tests wurden mit dem Chemilumineszenz-Kit zum Nachweis von IgG und IgM durchgeführt.

**Ergebnisse:**

88 (76,0%) der 116 Spender waren Männer, 28 (24%) Frauen. Das Alter der Blutspender verteilte sich wie folgt: 1,2% waren >40 Jahre, 4,3% 36–39 Jahre, 8,2% 31–35 Jahre, 19,1% 26–30 Jahre und 34,1% 18–20 Jahre alt. 75 Personen wurden positiv und 41 negativ auf IgG getestet, was eine CMV-Gesamtprävalenzrate von 64,6% ergibt. Keiner der 116 Blutspender wies eine CMV-IgM-Antikörperreaktivität auf. Die Prävalenz der IgG-Positivität war bei Personen im Alter von ≥26 Jahren höher. Von den IgG-seropositiven Personen waren 68 (64,76%) männlich und 7 (63,63%) weiblich. Der sozioökonomische Hintergrund bei Positivität war in 40% hoch, in 66,3% mittel und in 100% niedrig.

**Schlussfolgerung:**

Die Prävalenz deutet auf eine endemische Infektion hin, die möglicherweise mit Faktoren wie Alter, Geschlecht und sozioökonomischem Status zusammenhängt. IgM-Anti-CMV-Antikörper waren bei keinem Spender vorhanden. Aufgrund der hohen Kosten und des geringen Vorkommens an IgM-Antikörper-positiven Spendern kann das Screening auf Immunglobulin-M-Anti-CMV Antikörper daher auf Hochrisikorezipienten beschränkt werden. Leukozytendepletierte zelluläre Blutprodukte und die Auswahl von CMV-IgG- und CMV-IgM-negativen Spendern sind zwei prophylaktische Ansätze zur Senkung der Transfusionsübertragung (TT) von CMV. Somit unterstreicht unsere Studie die Bedeutung des CMV-Screenings in Blutbanken, die Notwendigkeit der Bewertung von Risikofaktoren für TT-CMV und die Notwendigkeit von Präventivmaßnahmen.

## Introduction

Blood transfusions are a vital component of medical treatment. On the other hand, transfusion-transmitted infections (TTI) pose possible risks. Therefore, blood collection, processing, and testing safety precautions to reduce the chance of transfusion-transmitted illnesses are important. The extent to which transfusion-transmitted diseases are a problem in a given country depends on the incidence of certain diseases. Precautions are taken to guarantee that blood transfusion therapy is safe for the intended population.

The cytomegalovirus (CMV), a member of the human herpes virus family, is spread by blood transfusions and is a major global health concern [1]. Seropositivity rates for CMV range from 60% to 100% in the population, making it a ubiquitous agent [2]. Among the most important infections that affect people with immunosuppression is CMV. As in the majority of cases with other herpes viruses, the patients survive their initial infection, while the virus remain dormant in the host for the rest of their lives [1]. However, in immunosuppressed individuals, these viruses can reactivate and cause a variety of clinical symptoms [3]. Rates of transmission from blood components to immunocompromised patients have been observed to reach up to 50% [4]. Therefore, the most effective method to lower the risk of CMV transmission would be to provide high-risk patients with blood products free of CMV. 

This study was carried out in response to the growing demand for cytomegalovirus-free blood products and to determine the prevalence of CMV antibodies among consenting blood donors. It is possible to determine the seroprevalence of cytomegalovirus among consenting blood donors, which may be helpful in determining whether CMV screening can shield high-risk individuals from infection. To date, there have been few such studies in India; thus, the current study was conducted to fill this knowledge gap. The results may assist health-sector policymakers and planners developing screening programs.

### Aim and objectives

To determine the seroprevalence of CMV among healthy, volunteer blood donors at the blood bank of a tertiary-care center by detecting immunoglobulin G, M and anti-CMV taking socio-economic and clinico-pathological parameters into consideration.

## Materials and methods

### Study design

This prospective cross-sectional study was carried out at the blood bank of the pathology department of Sree Balaji Medical College in Chromepet, Chennai, over the course of two years, from November 2022 to April 2024. A total of 116 volunteer blood donors were chosen. The ethics committee of Sree Balaji Medical College gave its approval. The minimum sample size required was n=116, as calculated by Dobson’s formula. 

### Collection of samples and antibody determination

5 mL of blood were extracted from each donor’s collecting bag and inserted into a sterile capped tube. The plasma was then separated and kept until needed, after which it was centrifuged.

IgG- and IgM-detection tests were performed with the chemiluminescence kit Electra (Goa, India). 

## Results

88 (76.0%) of the 116 donors were men, 28 (24%) were women. The age distribution of blood donors is shown in Table 1 (Tab. 1), blood group distribution in Table 2 (Tab. 2), and distribution according to SES in presented in Table 3 (Tab. 3). 

**Figure 1:**
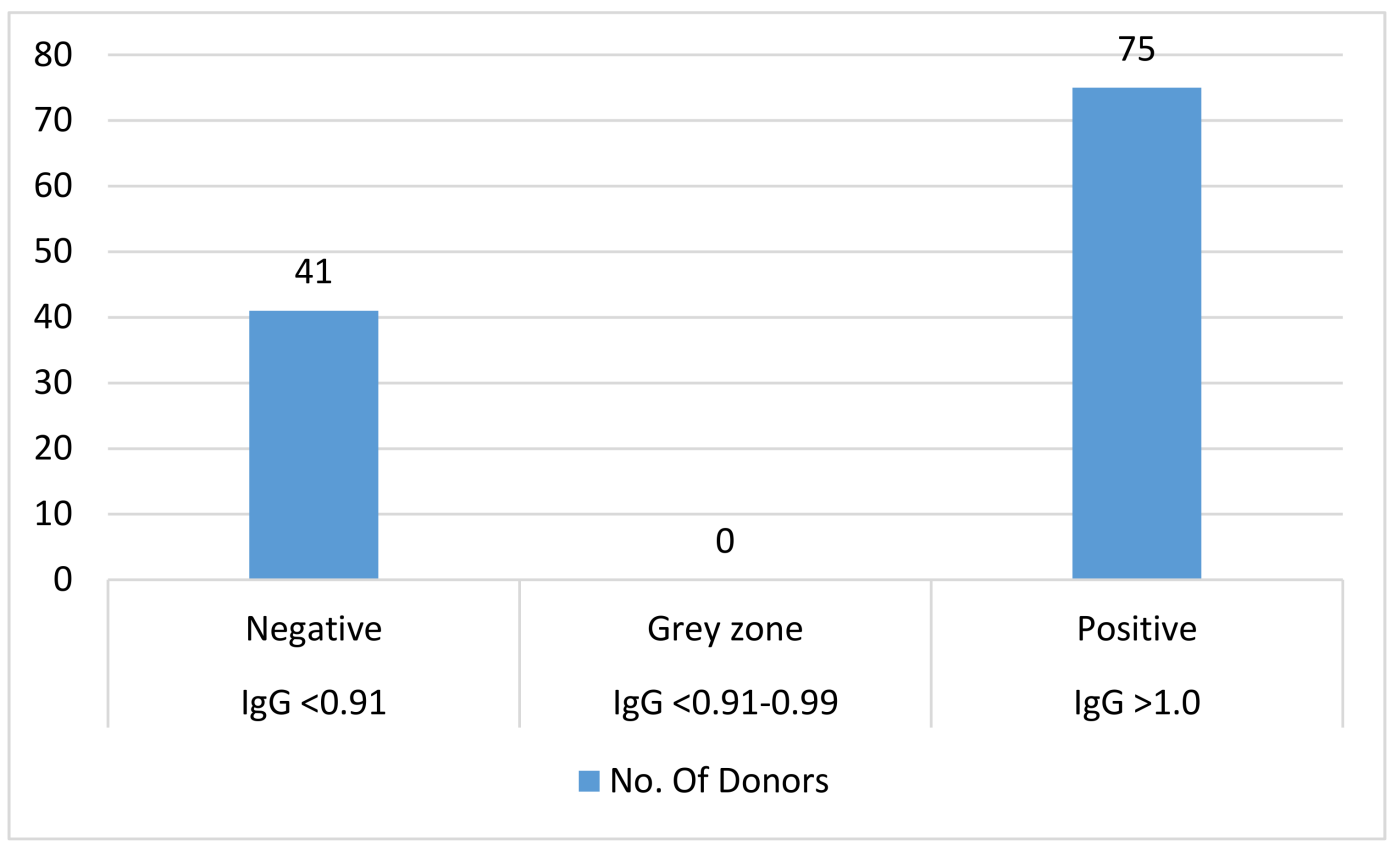
Bar diagram of titre results (CMV IgG by CLIA)

None of the 116 donors tested positive for the hepatitis B surface antigen (HbsAg). Based on IgG titres, 75 of the donors had a history of CMV exposure (Figure 1 (Fig. 1)), but none of the 116 blood donors were positive for IgM or had a indeterminate titre.

Table 4 (Tab. 4) shows an increased prevalence of IgG positivity among individuals aged ≥26 years (p<0.05).

Female were less frequently IgG seropositive (p<0.05) than males (Table 5 (Tab. 5)). The difference in seroprevalence between the sexes was statistically significant (p<0.05). 

Regarding IgG status, there were statistically significant (p<0.01) differences based on SES (Table 6 (Tab. 6)).

There was no discernible relationship found between CMV-IgG seropositivity and blood group (Table 7 (Tab. 7)).

## Discussion

CMV is a beta-DNA herpesvirus carried by almost 90% of the world’s adult population [5].

Given that voluntary blood donors are anticipated to supply the majority of blood transfusion needs, the current study aimed to identify the seroprevalence of CMV infection within this population. Therefore, the current study exclusively included voluntary blood donors because blood donations at our blood center are 100% voluntary.

In comparison with the IgG positivity of other studies, e.g., Seale et al. [6] (57%), Per Ljungman [7] (51.8%), and Amarapal et al. [2] (71%), our study showed 64.6% with positive IgG anti-CMV antibodies, which may indicate that they had previously been exposed. This is comparable with the percentages of industrialized nations.

In agreement with our study, Ibramin et al. [8] also showed that voluntary blood donors were more prevalent in the age groups of 20–40 years and were predominantly male. Many females in our country are anemic, which prevents them from donating blood; this would account for the male predominance among blood donors.

According to the updated Modified Kuppuswamy socioeconomic status scale, our donors were classified as upper, middle, or lower class. The majority of our volunteers (73.0%) were of middle SES, followed by lower SES (14.1%), with the fewest being of SES (13.1%). 

An IgG value of <0.91 is considered negative, showiing a lack of previous exposure to cytomegalovirus. If the values are between 0.91 and 0.99 (inconclusive), re-testing is necessary; titre values >1.0 (positive) mean that the donor has had a history of CMV exposure. When the IgM value is <0.90 (negative), it shows a lack of previous exposure to cytomegalovirus. Values between 0.91 and 1.1 (inconclusive) indicate that re-testing is necessary. Titre values >1.1 (positive) show that the donor has had a history of CMV exposure. Based on these values, 75 of 116 donors showed a higher titre value of IgG and 41 showed negative results for IgG. Also, all 116 samples showed a negative titre for IgM. 

The IgG anti-CMV antibody test is mainly done to reveal the patient’s past infections.

In our study, IgG serology was positive mostly among donors in their late twenties. Statistically significant differences in CMV IgG status were found between the different age groups (p<0.05). The study by Nikolich-Zurich and van Lier [4], demonstrated that when individuals who are long-time positive for IgG anti-CMV antibodies age, it leads to an increased rate of mortality among these individuals. Another factor that explains these higher death rates is that aging and cytomegalovirus both reduce innate and adaptive immunity, which further enhances the risk of mortality for seropositive individuals as they age [9]. Age-related changes to the T-cell immune system have even been attributed to cytomegalovirus infection as the underlying reason of morbidity [10].

Our study is consistent with research done by Koch et al. [10], who found that as people age, the prevalence of CMV antibodies rises. They reported that the prevalence of the antibody increased from 81% among 21- to 30-year olds to 88% between the ages of 41 to 50 years.

Nearly 30% of our donors in our study were in the age group of 18 to 20 years among them, nearly 50% were IgG positive. This can be reduced by proper guidance on the importance of screening and early detection of cytomegalovirus. Future research should assess exposure and personal behaviour in younger adults prior to a vaccination intended to prevent congenital CMV infection, as half of the young adults in our study had already contracted the virus [11].

Since cytomegalovirus prevalence seems to occur in any age group (younger, middle, or older) and does not seem gender-specific, it is beneficial to include the screening for cytomegalovirus along with other transfusion-transmitted infection screening, so that the recipients of the blood will not contract the virus. 

A little over 40% of donors with higher SES tested positive for cytomegalovirus, compared to 66% from middle and 100% from lower SES groups. This concurs with a study done in Finland by Mustakangas et al. [12], which found that seropositivity more frequent in lower socioeconomic groups than in higher socioeconomic groups. According to a study done in Punjab [13], seropositivity decreased as socioeconomic status increased. It is well known that individuals from low socioeconomic backgrounds will have less awareness about the risk of cytomegalovirus transmission, and due to their lifetime exposure to these illnesses, people from lower socioeconomic backgrounds tend to have higher antibody levels [14]. Another study also showed that lower-SES pregnant women from Norway had a higher rate of positive serology for immunoglobulin G cytomegalovirus than did pregnant women with higher SES [15]. Thus, the present study strengthens the evidence that SES plays an important role in the prevalence of cytomegalovirus among the general population. More awareness should be created about screening for cytomegalovirus among the lower SES population. Low SES is associated with increased exposure to cytomegalovirus due to factors such as larger family sizes and living in crowded conditions. Analogous to the literature, this study found no discernible relationship between CMV IgG seropositivity and blood group. 

The IgM anti-CMV antibody test is mainly done to determine the presence of an acute infection in the individual. None of the donors in our study had a positive IgM anti-CMV antibody test, proving that there was no initial infection. Our IgM anti-CMV seropositivity rates matched those of studies conducted in Ghana by Adjei et al. [16], in Chandigarh by Pal et al. [17], and in New Delhi by Kothari et al. [18]. In a developing country like India, routine IgM antibody testing may not be possible as the seropositivity rates arevery low, and the cost is high.

In tertiary care hospitals, it is recommended that blood units transfused to newborns, organ transplant recipients, patients with malignancies, and other immunocompromised patients undergo anti-CMV IgM testing or implement preventive strategies to reduce CMV infection.

Due to the high seropositivity of anti-IgG CMV, discarding blood positive for anti-IgG CMV is not feasible, but blood screened positive for anti-IgM CMV is recommended to be discarded.

Out of 116 blood donors in our study, none had a HbAg-positive result. According to a study by Bayram et al. [19], individuals with chronic hepatitis B and hepatitis C had a higher incidence of cytomegalovirus infection. Nearly 50% of patients with chronic HBV and 36% of individuals with chronic HCV had evidence of CMV infection.

Blood transfusion has long been cited as a risk of CMV, notably in immunocompromised individuals (e.g., organ transplant recipients) and hospitalized neonates and infants. Nonetheless, the risk of transfusion-transmitted CMV remains contentious, given that a high proportion of the general population is already CMV seropositive, and most infections are asymptomatic.

Donor/recipient CMV serological status remains the main risk factor influencing the incidence and mortality of CMV disease after transplantation. Proper selection of donor and recipient, regular and careful monitoring, early intervention in CMV reactivation, and rapid and effective treatment when the disease develops remain crucial to decreasing the risk of post-transplantation CMV reactivation or disease.

Measures to reduce the risk of TT-CMV have been evaluated in clinical studies, including leucocyte depletion of cellular blood products and/or the selection of CMV-IgG-negative donations.

It is not practical to discard blood that tests positive for IgG anti-CMV antibody due to the 64.6% seropositivity in our study. When seronegative blood is not available, substitutes such as leukoreduced blood products can be used. For individuals who are more susceptible to cytomegalovirus illness, leukoreduced components should nonetheless be utilized less frequently than cytomegalovirus seronegative components when it comes to transfusion requirements.

## Conclusion

The high prevalence 64.7% of CMV in our study points to an endemic infection, which was related to factors such as age, gender, and socioeconomic status. 

IgM anti-CMV antibodies were absent in every donor. Therefore, because of the high cost and low prevalence of IgM anti-CMV positivity among blood donors, IgM anti-CMV antibody screening can be limited to only high-risk, susceptible recipients.

The prophylactic approaches to lower the seroprevalence of TT-CMV include the use of leukocyte-depleted cellular blood products and the selection of CMV-IgG and CMV-IgM-negative donors for the benefit of the recipients.

Thus, our study highlights the importance of screening for CMV in addition to the routine screening tests at blood banks, the need to assess risk factors for TT-CMV, and the need to take preventive action.

## Notes

### Ethical approval

The Sree Balaji Medical College’s ethics committee in Chromepet, Chennai, gave its approval.

### Competing interests

The authors declare that they have no competing interests.

## Figures and Tables

**Table 1:**
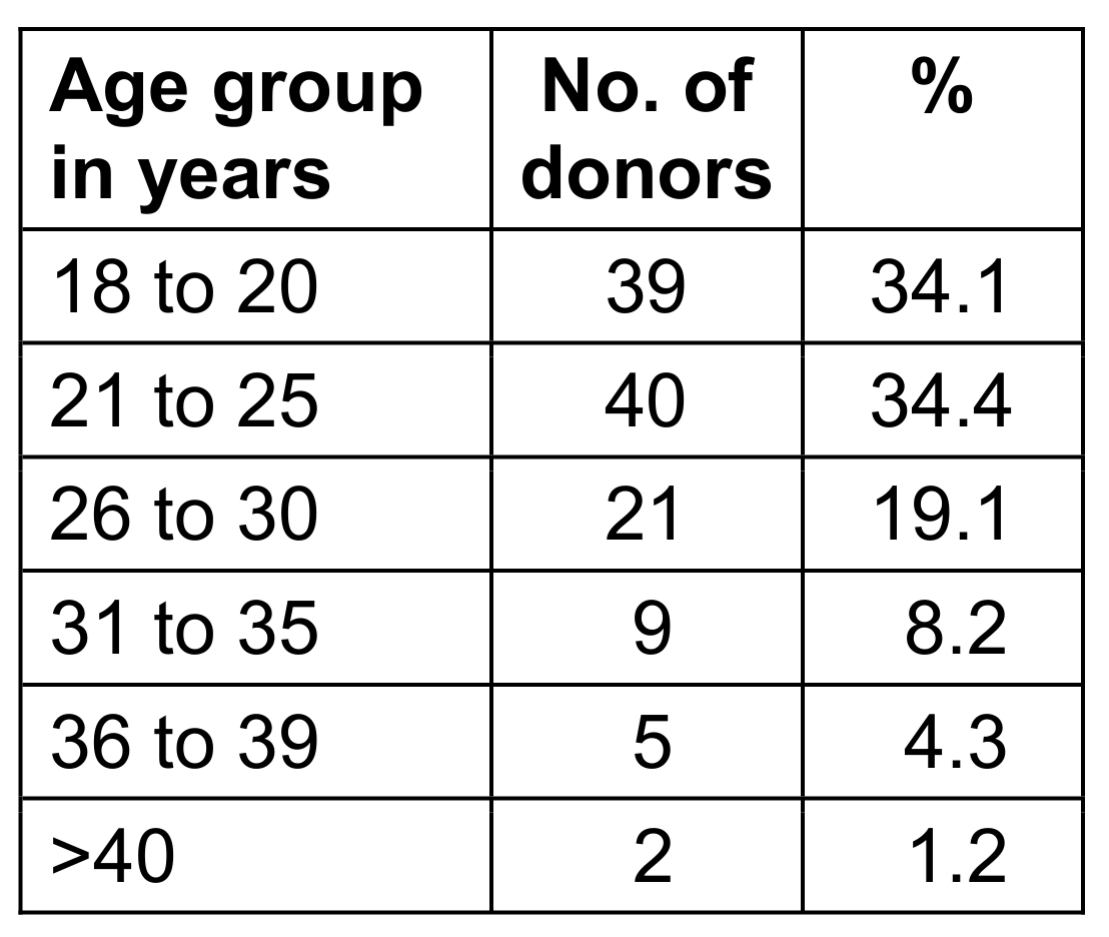
The study group’s age distribution

**Table 2:**
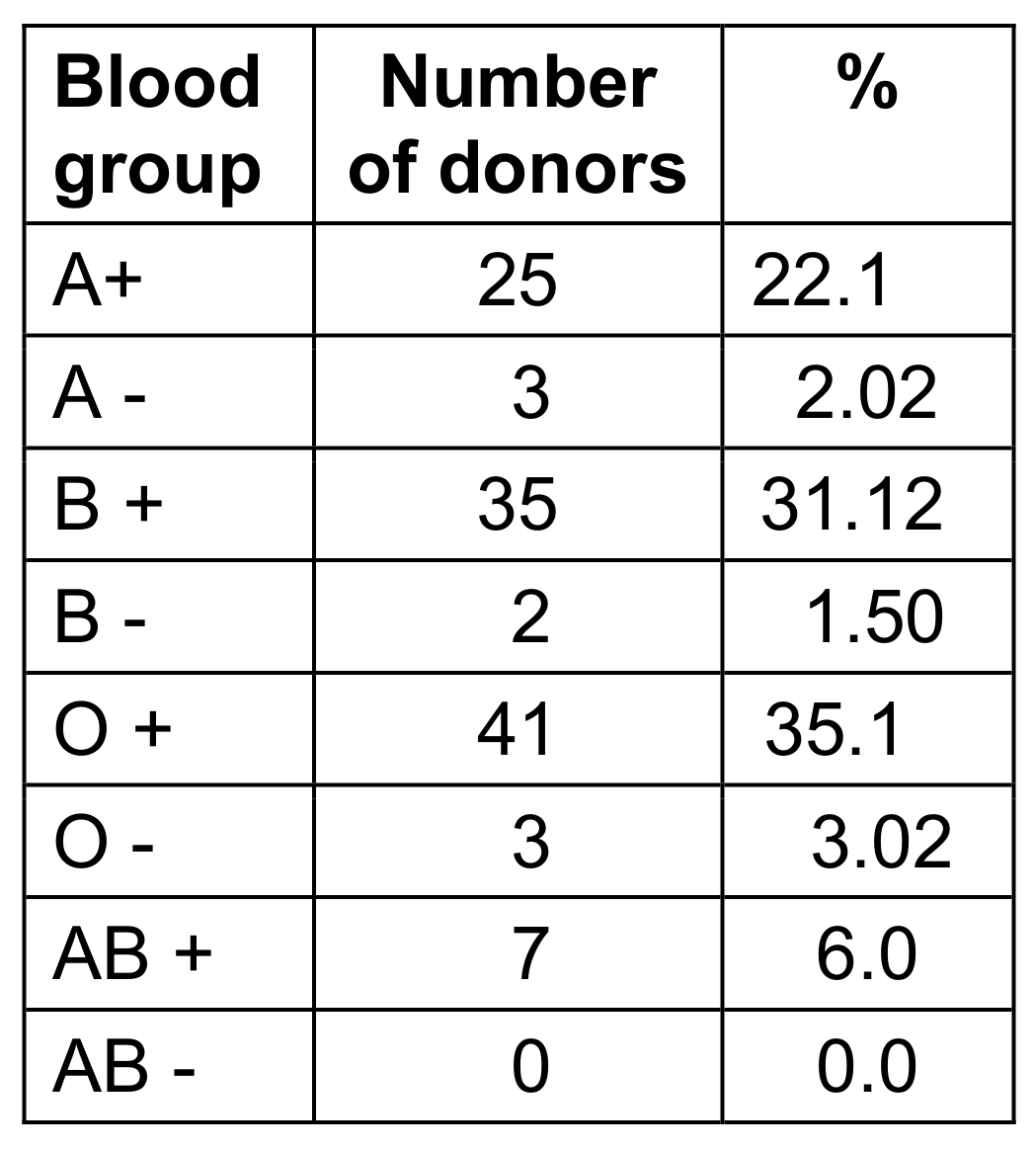
The blood group’s distribution

**Table 3:**
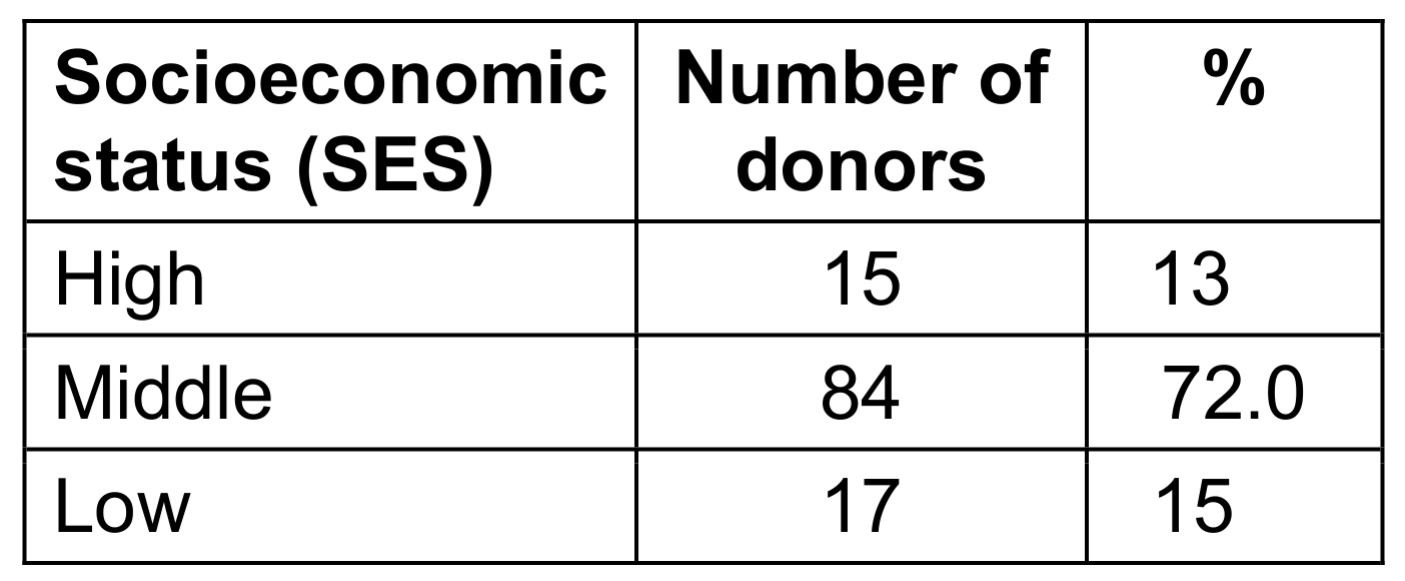
Distribution according to SES

**Table 4:**
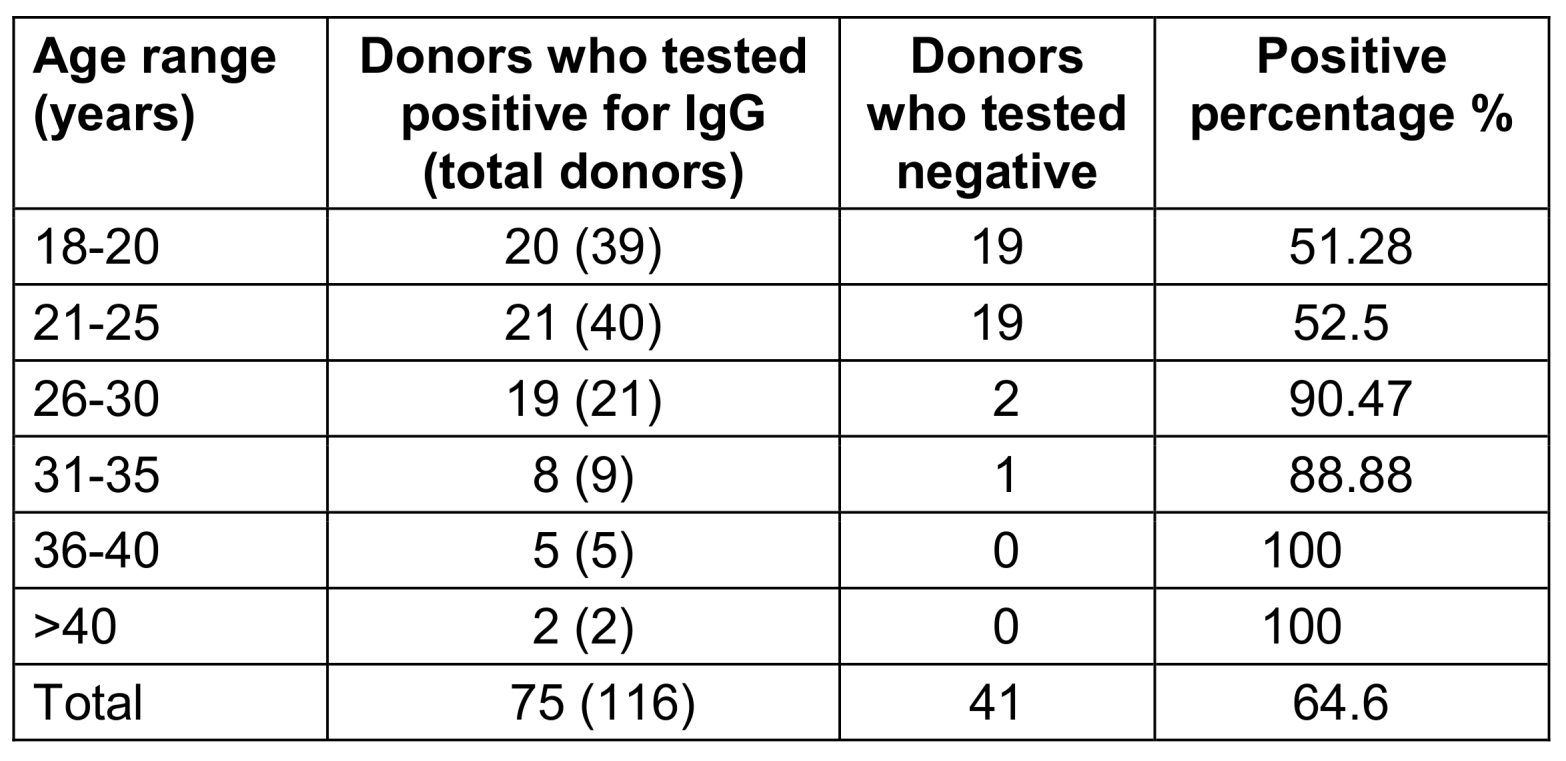
Age range of IgG positive individuals

**Table 5:**
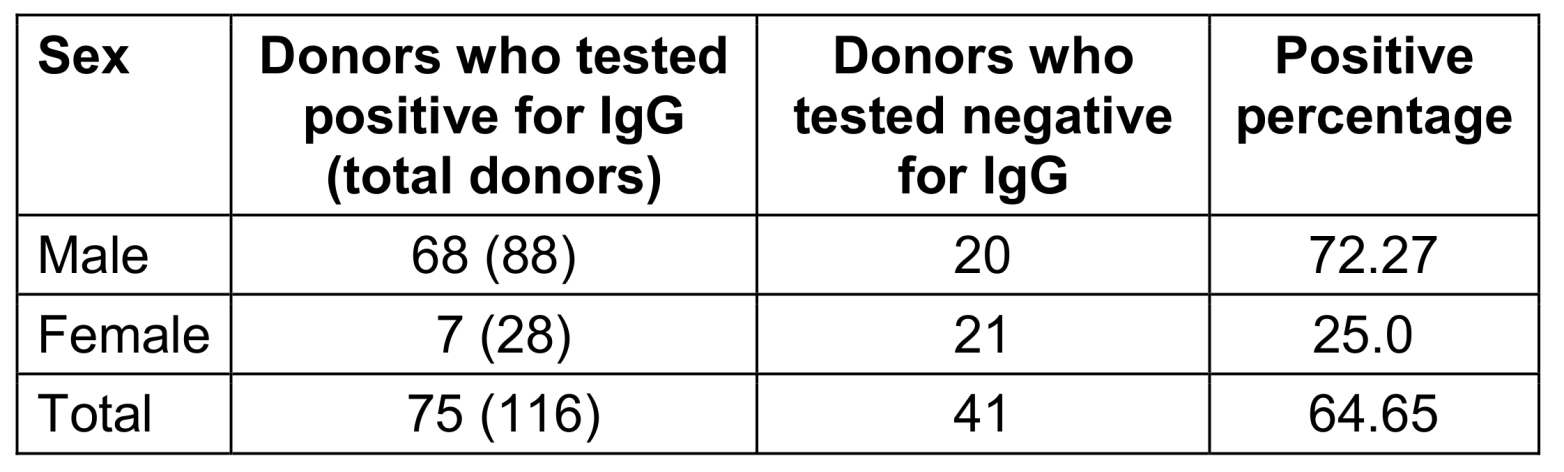
Prevalence of IgG seropositivity by gender

**Table 6:**
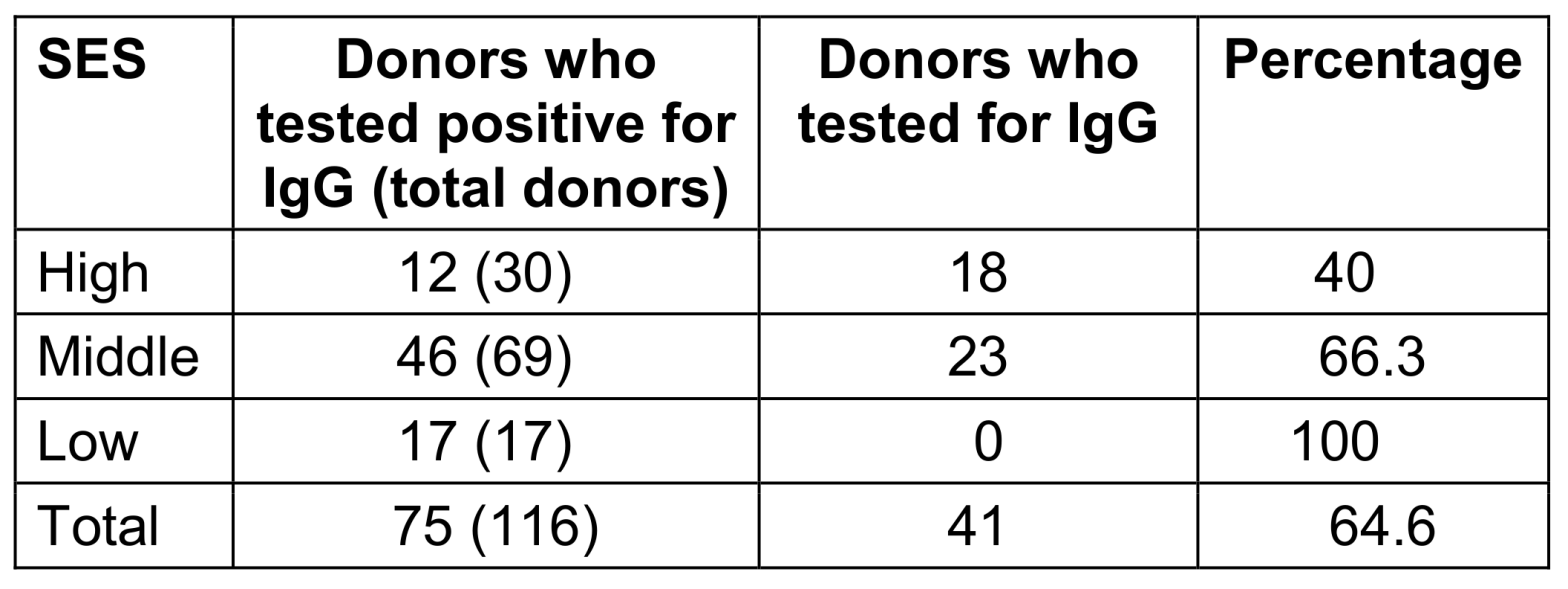
Socioeconomic status (SES) of IgG-seropositive donors

**Table 7:**
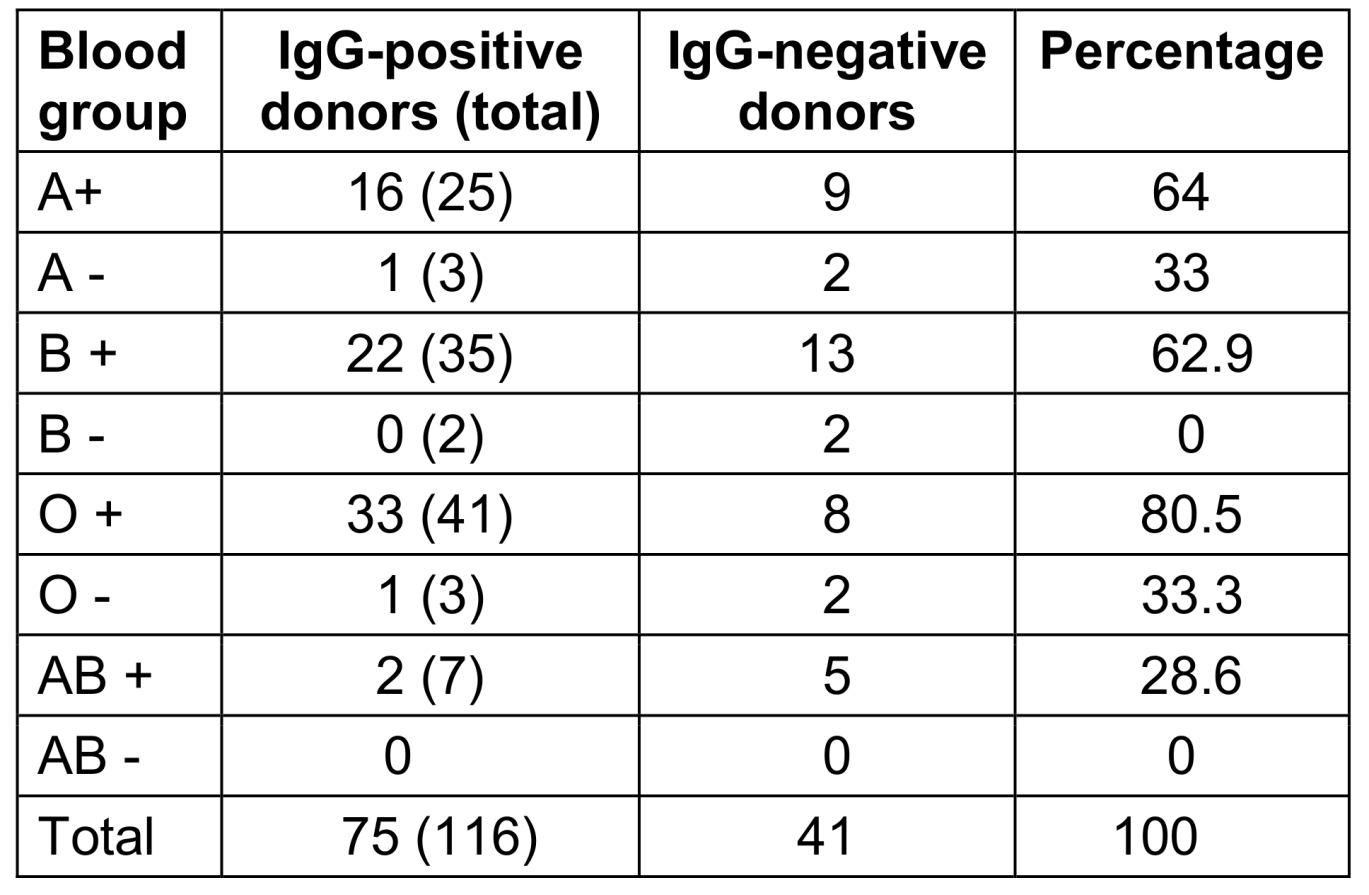
Blood groups of IgG-seropositive donors
